# Non-Isothermal Analysis of the Crystallization Kinetics of Amorphous Mg_72_Zn_27_Pt_1_ and Mg_72_Zn_27_Ag_1_ Alloys

**DOI:** 10.3390/ma17020408

**Published:** 2024-01-13

**Authors:** Aleksandra Pierwoła, Janusz Lelito, Halina Krawiec, Michał Szucki, Łukasz Gondek, Tomasz Kozieł, Rafał Babilas

**Affiliations:** 1 Faculty of Foundry Engineering, AGH University of Krakow, 30 Mickiewicza Street, 30-059 Krakow, Poland; pierwola@agh.edu.pl; 2 Foundry Institute, Technische Universität Bergakademie Freiberg, 4 Bernhard-von-Cotta-Str., 09599 Freiberg, Germany; michal.szucki@gi.tu-freiberg.de; 3 Faculty of Physics and Applied Computer Science, AGH University of Krakow, 30 Mickiewicza Street, 30-059 Krakow, Poland; lgondek@agh.edu.pl; 4 Faculty of Metals Engineering and Industrial Computer Science, 30 AGH University of Krakow, Mickiewicza Street, 30-059 Krakow, Poland; tkoziel@agh.edu.pl; 5 Department of Engineering Materials and Biomaterials, Silesian University of Technology, 18a Konarskiego Street, 44-100 Gliwice, Poland; rafal.babilas@polsl.pl

**Keywords:** metallic glass, Mg_72_Zn_27_Pt_1_, Mg_72_Zn_27_Ag_1_, crystallization kinetics

## Abstract

In this study, thin ribbons of amorphous Mg_72_Zn_27_Pt_1_ and Mg_72_Zn_27_Ag_1_ alloys with potential use in biomedicine were analyzed in terms of the crystallization mechanism. Non-isothermal annealing in differential scanning calorimetry (DSC) with five heating rates and X-ray diffraction (XRD) during heating were performed. Characteristic temperatures were determined, and the relative crystalline volume fraction was estimated. The activation energies were calculated using the Kissinger method and the Avrami exponent using the Jeziorny–Avrami model. The addition of platinum and silver shifts the onset of crystallization towards higher temperatures, but Pt has a greater impact. In each case, $E_{g}$ > $E_{x}$ > $E_{p}$ (activation energy of the glass transition, the onset of crystallization, and the peak, respectively), which indicates a greater energy barrier during glass transition than crystallization. The highest activation energy was observed for Mg_72_Zn_27_Pt_1_ due to the difference in the size of the atoms of all alloy components. The crystallization in Mg_72_Zn_27_Ag_1_ occurs faster than in Mg_72_Zn_27_Pt_1_, and the alloy with Pt has higher (temporary) thermal stability. The Avrami exponent (*n*) values oscillate in the range of 1.7–2.6, which can be interpreted as one- and two-dimensional crystal growth with a constant/decreasing nucleation rate during the process. Moreover, the lower the heating rate, the higher the nucleation rate. The values of *n* for Mg_72_Zn_27_Pt_1_ indicate a greater number of nuclei and grains than for Mg_72_Zn_27_Ag_1_. The XRD tests indicate the presence of α-Mg and Mg_12_Zn_13_ for both Mg_72_Zn_27_Pt_1_ and Mg_72_Zn_27_Ag_1_, but the contribution of the Mg_12_Zn_13_ phase is greater for Mg_72_Zn_27_Ag_1_

## 1. Introduction

Due to their high strength and plastic properties, magnesium–zinc alloys are used in many areas [1,2]. Additionally, due to their biocompatibility with the human body, they seem to be an ideal candidate for biomedical materials [1,2,3]. Unfortunately, surrounded by body fluids, the classic magnesium–zinc alloy with a crystal structure undergoes rapid corrosion, i.e., degradation, in the human body [1]. The biodegradability phenomenon of magnesium–zinc alloys can be positively used in temporary implant treatments (for bone adhesion, for example), which would eliminate the need for implant removal surgery [2]. The corrosion process, however, must be slowed down and somehow controlled.

Corrosion resistance can be improved by adding highly resistant alloying elements [3]. They must, however, also be biocompatible and biodegradable. There is also a possibility of turning an alloy into a metallic glass with a disordered and amorphous structure, where the lack of grains will significantly slow the corrosion process [2,3]. This relatively new group of materials is gaining more and more interest among scientists [4,5]. The amorphous nature of metallic glasses gives the material better corrosion resistance and better mechanical properties than crystalline materials. The low plasticity of amorphous alloys limits their wide application [6]. Therefore, the partial crystallization process of amorphous alloys may be a solution that can improve the plastic properties while slightly reducing the corrosion properties.

Additionally, it should be taken into account that the amorphous form of the alloy is metastable, i.e., the alloy will crystallize over time, especially with increasing temperature [5]. The phenomena of crystallization and corrosion of metallic glasses occur at different rates, depend strictly on the chemical composition, and are still not well understood [1]. It is therefore important to understand the crystallization kinetics of metallic glasses based on Mg alloys.

The crystallization process in metallic glass can be analyzed using, among others, a differential scanning calorimeter (DSC) [7]. Next, models popular in the literature, such as Johnson–Mehl–Avrami [8,9] and Kissinger [10], are used for the interpretation of energy values from DSC experiments. Therefore, it is possible to calculate the kinetic parameters and draw conclusions about the stability of glass, as well as the rate and nature of crystallization (the nucleation and growth of crystals at different stages of the process) [7,11]. The improper use of a model or rate constant in experimental data can, however, lead to physically meaningless or, worse still, misleading results. Before using the models in kinetic analysis and determining the process rate constants, it is necessary to consider the phenomena occurring during the crystallization process, the temperature program used during the experiment (isothermal or non-isothermal), and the nature of the material being tested (alloy, polymer, etc.).

In general, systems of three or more components were observed to undergo glass transition more easily than binary systems [11]. Various Mg-Zn-based metallic glasses have been analyzed in the literature, but these with the addition of Ca are the most popular and investigated ones [1,2,4,12,13,14,15,16,17]. As mentioned, a small change in the chemical composition can significantly affect the strength parameters, the ability to undergo glass transition, and the crystallization mechanism [4].

**Figure 1:**
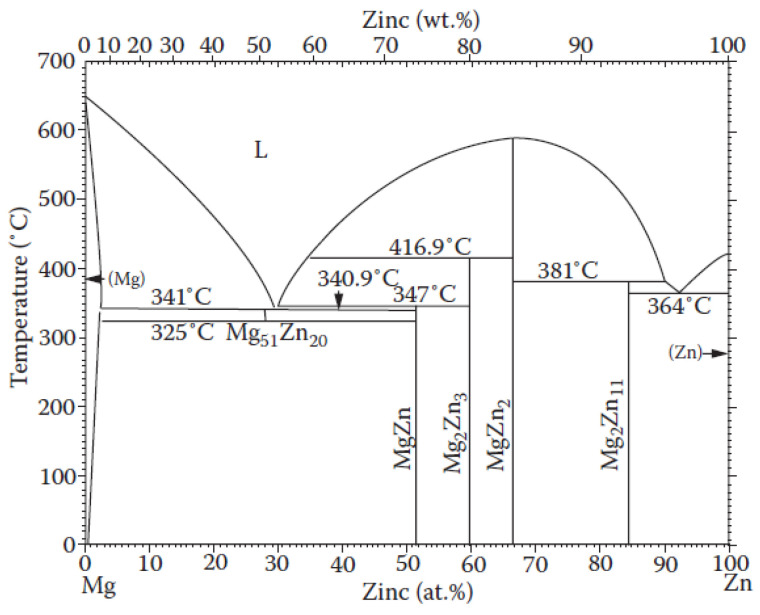
Phase diagram of the Mg-Zn alloy [18].

This article aims to compare the crystallization kinetics (using DSC) and the analysis of the phase composition (using XRD) during non-isothermal annealing for two potentially biodegradable metallic glasses with a chemical composition that is approximately eutectic—Mg_72_Zn_27_Pt_1_ and Mg_72_Zn_27_Ag_1_. It is obvious that alloys with a chemical composition close to the eutectic point can be easily transformed into metallic glasses (Figure 1). The transformation of the alloy into a metallic glass depends on the cooling rate of the liquid alloy. Alloys whose chemical composition differs significantly from the eutectic point require higher cooling rates. The presence of platinum and silver in these alloys may help facilitate the glass transition of these alloys. In terms of toxicity, both platinum and silver are non-toxic. There is no information in the literature on the crystallization of amorphous Mg-Zn alloys with the addition of platinum. This may be related to the large difference in melting point between these ingredients and platinum. Moreover, magnesium and zinc have a high affinity for oxygen, which creates problems when producing such alloys.

## 2. Materials and Methods

The initial alloys in the form of ingots were prepared using induction melting under an argon atmosphere with the total chemical formulas Mg_72_Zn_27_Pt_1_ and Mg_72_Zn_27_Ag_1_ (% by atomic). The chemical composition of both alloys was approximately eutectic. Magnesium, zinc, silver, and platinum with a purity of 99.9% were used. Amorphous ribbons of these two alloys were cast using a rapid solidification method—melt spinning. Using this technique, 150 μm thick ribbons were obtained. The melting and casting processes took place under argon protection. The peripheral speed of the wheel was 40 m/s. The amorphous nature of the resulting samples was confirmed using X-ray diffraction. Then, using a differential scanning calorimeter (TA DSC Q20, Eschborn, Germany), each alloy was heated non-isothermally from room temperature to approximately 550 °C at five heating rates: 5, 10, 20, 40, and 80 K/min. A high purity argon flow was used.

Using high-temperature X-ray diffraction (HT-XRD), the samples were heated in the temperature range of 300–700 K with a 5 K/min heating rate. Results were recorded every 10 K and the appearance of peaks coming from the emerging crystalline phase was observed. The location of peaks at specific places allows for the identification of crystallizing phases. The X-ray diffraction studies were carried out using a Panalytical Empyrean (Almelo, Nederland), diffractometer equipped with a Cu Kα X-ray source. The non-ambient temperature studies were performed in an Anton Paar HTK 1200N chamber. The sample position was corrected for thermal displacement, and the temperature stabilization was better than 0.2 K.

## 3. Results and Discussion

**Figure 2:**
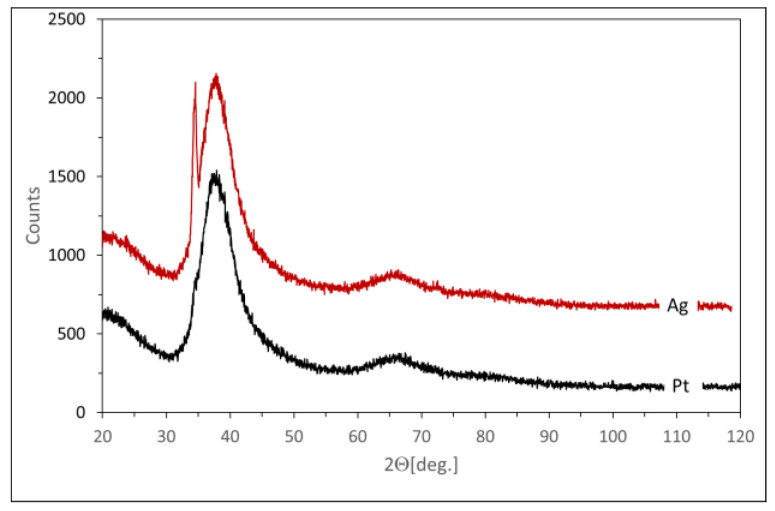
XRD-patterns of the investigated alloys at room temperature.

The samples of the fabricated ribbons of the two analyzed alloys were investigated for preliminary X-ray diffraction testing to confirm their amorphousness. The X-ray diffraction pattern of the alloy with Pt confirmed complete amorphousness (Figure 2). In turn, the Ag ribbons contained a very small part of Mg_51_Zn_20_ and Mg phases, respectively. An additional quantitative analysis was therefore carried out. For this purpose, the ribbon was powdered. The XRD pattern of the powdered ribbon at 300 K is shown in Figure 1 and marked “Ag”. The level of crystallinity was estimated to be no higher than 2.5% and is acceptable in terms of amorphousness for further investigation. The crystalline fraction of the powdered ribbon was estimated using High Score Plus software (version 3.12). The procedure was as follows:

- The background resulting from the scattering of X-rays in air and the optics of the incident and diffracted beam (slits/collimators) were measured.
- The sample was then measured in the same way. The optics of the incident beam were set to illuminate only the sample surface and exclude the signal from the fixture.
- A background scan was then extracted from the sample scan, and a procedure was used to compare the surface under the reflections with the total surface. This procedure can be used because, according to EDS data, the amorphous and crystalline phases have the same composition. Therefore, no correction was required for different scattering lengths. The maximum amorphous contribution corresponds to interplanar distances of 2.9–1.9 Å. This correlates quite well with the expected metallic radius of Mg, Zn, and Ag, which are 1.6, 1.37, and 1.75 Å, respectively.

**Figure 3:**
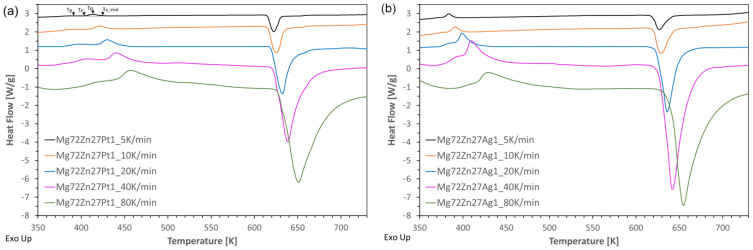
DSC curves for the (**a**) Mg_72_Zn_27_Pt_1_ and (**b**) Mg_72_Zn_27_Ag_1_ alloys heated at the rate of 5, 10, 20, 40, and 80 K/min.

The non-isothermal heating of metallic glasses in DSC at different rates *β* (5, 10, 20, 40, and 80 K/min) allowed for obtaining thermograms, heat flow H vs. temperature T (Figure 3). It should be noted that each curve shows a small endothermic peak corresponding to the glass transition, followed by an exothermic reaction that is characteristic of the crystallization process. Additionally, at the end of each curve, an endothermic peak is visible, which is related to the melting process. Characteristic temperatures were determined for each curve: the glass transition $T_{g}$, the onset of crystallization $T_{x}$, the peak $T_{p}$, and the end of crystallization $T_{x\_end}$ temperatures. These temperature values are presented in Table 1. The characteristic temperatures are strictly dependent on the heating rate. As the heating rate increases, $T_{g}$, $T_{x\_end}$, and $T_{p}\;$shift towards higher temperature values. This phenomenon is related to the kinetics of glass transition and crystallization. This is due to the fact that the nucleation process is thermally activated, while the rate dependence of the kinetic glass transition results from relaxation processes in the glass transition region [19]. Additionally, the faster the heating rate, the wider and higher the crystallization peak.

The crystallization process occurs after a certain period from the beginning of heating called incubation time *τ*. The higher the heating rate, the faster the temperatures needed to initiate crystallization are reached, which leads to shorter incubation times for higher rates.

Moreover, the addition of alloying elements, i.e., Pt or Ag, to the base alloy Mg_72_Zn_28_ [20] causes their shift towards higher temperatures. Platinum has the greatest impact (it raises $T_{x}$ by approximately 50 K), because it has a higher melting point (1770 °C/2043 K) [21].

The relative crystallized volume at current temperature for each curve was determined using the following equation [22]

(1) $$x=\frac{\int_{T_{x}}^{T}\frac{dH}{dT}dT}{\int_{T_{x}}^{T_{x\_end}}\;\frac{dH}{dT}dT}$$

**Figure 4:**
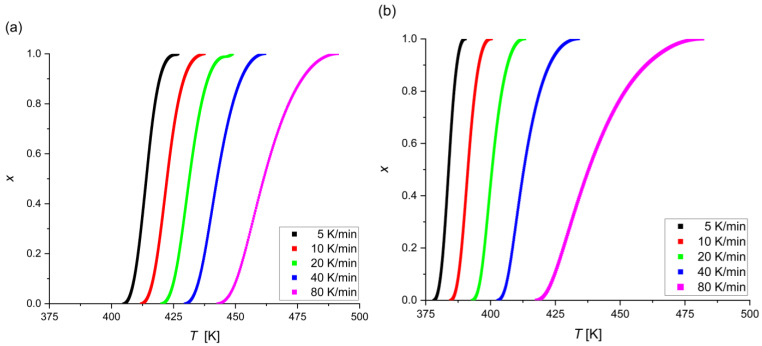
Relative crystallized volume versus temperature at different heating rates for the (**a**) Mg_72_Zn_27_Pt_1_ alloy and the (**b**) Mg_72_Zn_27_Ag_1_ alloy.

The graphical representations of the dependence *x*(*T*) for each alloy and heating rate are shown in Figure 4. These curves have a typical sigmoidal shape. For both amorphous alloys, an increase in the heating rate causes the beginning and end of the transformation to shift towards higher temperature values. Additionally, it can be noticed that the alloy containing platinum compared to the alloy containing silver has the beginning and end of the transformation shifted towards higher temperature values.

**Figure 5:**
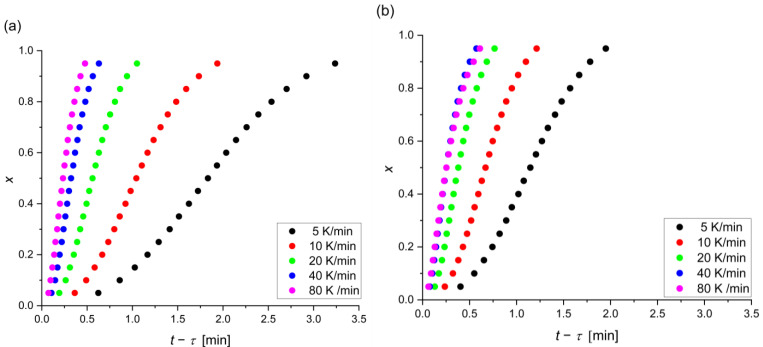
Relative crystallized volume versus time (*t* − *τ*) at different heating rates for the (**a**) Mg_72_Zn_27_Pt_1_ and (**b**) Mg_72_Zn_27_Ag_1_ alloys.

The current time of crystallization (*t* − *τ*) was read for every 0.05 crystallized volume. Figure 5 shows the relationship between the relative crystallized volume and time for each alloy and heating rate. Heating at the slowest rate takes the longest time and has the lowest temperature range. As can be seen, crystallization for the alloy with Ag is faster than Pt, which suggests the greater thermal stability of Mg_72_Zn_27_Pt_1_.

**Figure 6:**
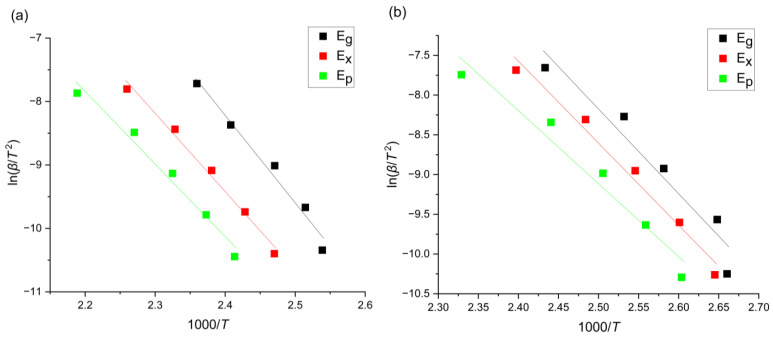
Kissinger plots for the (**a**) Mg_72_Zn_27_Pt_1_ alloy and the (**b**) Mg_72_Zn_27_Ag_1_ alloy.

Using the Kissinger method, the activation energies for the glass transition $E_{g}$, the onset of crystallization $E_{x}\;$and the crystallization peak $E_{p}$ were calculated through the following equation [10]

(2) $$ln\left(\frac{\beta }{T^{2}}\right)=-\frac{E}{RT}+\ln\left(\frac{k_{0}R}{E}\right)$$

where R is a universal molar gas constant 8.31 [J/molK] and $k_{0}$ is a reaction constant. Plotting $\ln\left(\beta /T^{2}\right)$ versus 1000/T allowed for the reading of energy values (compared in Table 2) from the slope of the appropriate curves (Figure 6).

In each case, $E_{g}$ is higher than $E_{x}$ and $E_{p}$, which indicates a greater energy barrier during glass transition than crystallization. The highest values of activation energy at all characteristic points were observed for the alloy with Pt. This may be due to the difference in the size of the atoms of the alloy components. The greater the difference in the sizes of atoms of individual elements, the more difficult it is to arrange them in the structure. According to the size of the atoms, the atomic radius (van der Waals) of Mg equals 170 pm and that of Zn 139 pm [23]. For comparison, the atomic radius of Pt and Ag equals 209 pm and 172 pm, respectively. The size of the atomic radius of Pt differs significantly from that of Mg and Zn, which can improve thermal stability.

Additionally, the difference between $E_{g}$ and $E_{x}$ is greater for Mg_72_Zn_27_Pt_1_. Furthermore, this manifested itself earlier (Figure 3a) as the larger distance between $T_{g}$ and $T_{x}$ in the thermogram for the Pt alloy.

The average Avrami exponent n and reaction rate constant k were calculated for both alloys and all heating rates using the Avrami model (Expressions (3) and (4)) [8,9,24,25]. Because of non-isothermal conditions, the *k* should be converted using the Jeziorny method and only $k_{c}\;$is used for analysis (Equation (5)) [25,26].

(3) $$x=1-\exp(-(k{t)}^{n})$$

(4) ln⁡−ln⁡(1−x)=nln⁡k+n ln⁡t

(5) $$\ln k_{c}=\frac{\ln k}{\beta }$$

**Figure 7:**
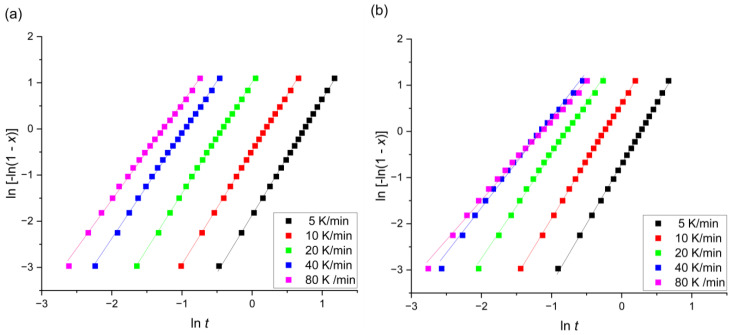
Jeziorny–Avrami plot at different heating rates for the (**a**) Mg_77_Zn_22_Pt_1_ and (**b**) Mg_77_Zn_22_Ag_1_ alloys.

After plotting $\ln\left[-\ln\left(1-x\right)\right]$ versus ln⁡t, it was possible to calculate the *n* from the slope and *k* ($k_{c}$) from the intercept of curves in Figure 7.

The *n* values allow for the interpretation of the crystallization mechanism in the investigated alloys. It is assumed that transformations in solids are controlled by diffusion. According to the literature [26,27], *n* = 1 means surface nucleation; 1 < *n* < 1.5 means the growth of large-volume particles; *n* = 1.5 means crystal growth with a nucleation close to zero; 1.5 < *n* < 2.5 means crystal growth with a decreasing nucleation rate; *n* = 2.5 means crystal growth with constant nucleation rate; and *n* > 2.5 means crystal growth with an increasing nucleation rate.

Furthermore, in a structure without previous nuclei, the coefficient responsible for the growth dimensionality is equal to *m* = *n* − 1. The *m* = 3 is for the three-dimensional, *m* = 2 for the two-dimensional, and *m* = 1 for the one-dimensional growth of crystal particles [26,27].

Based on the above information, it can be said that crystallization in the tested alloys is mainly based on one- and two-dimensional crystal growth. Crystal growth in two dimensions is characteristic of thin films [27].

**Figure 8:**
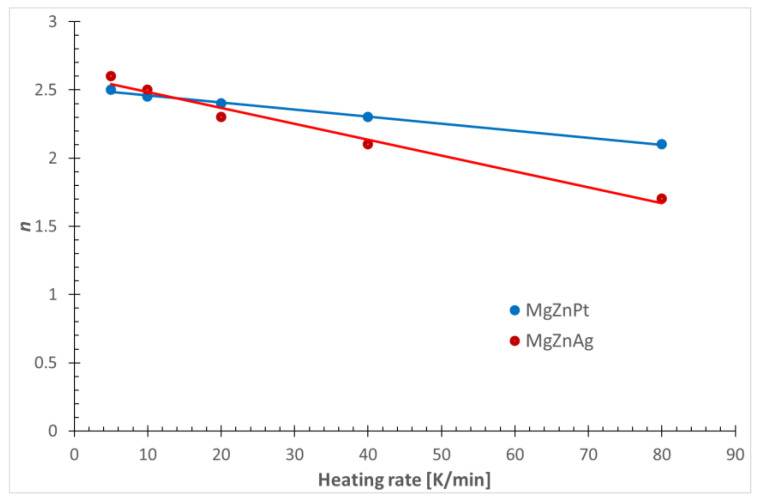
Avrami exponent as a function of cooling rate.

The results in Table 3 show that in the case of the Avrami exponent, the obtained values are not integer. The frequent occurrence of such values can be explained in many ways in practice. Typically, explanations are based on models of the nucleation mechanism. In the case of the above work, the interpretation of the results must take into account the specificity of the non-isothermal crystallization process. This specificity results from the fact that the only parameter that changes significantly in subsequent measurements is the heating rate of the amorphous ribbon. It can therefore be concluded that the heating rate is one of the factors responsible for the variation in the value of the Avrami exponent *n*. The proof of the validity of such an assumption is the linear nature of the graph illustrating the dependence of the Avrami exponent on the heating rate, as shown in Figure 8.

The heating rate values corresponding to the Avrami exponent values can be determined from this graph by extrapolation. It was found that the value of *n* = 2.55 and 2.65, respectively, for an alloy containing platinum and silver, is characteristic of a heating rate of zero, i.e., isothermal crystallization conditions.

Figure 8 shows that the values of *n* < 2.55 and 2.65 are characteristic of the non-isothermal crystallization range, *β* > 0. Therefore, the faster the heating, the lower the value of n. It seems that this relationship is directly related to the increasing effect of the athermal nucleation process, which is associated with an increase in the heating rate.

The *n* value for these alloys decreases with increasing heating rate. This can be explained by faster crystallization, i.e., insufficient time needed for equal crystal growth in all directions. Furthermore, the calculated *n* values oscillate in the range of 1.7–2.6, which can be interpreted as a constant and decreasing nucleation rate throughout the process. Generally, the lower the heating rate, the higher the nucleation rate. But, comparing the *n* values for each alloy at a specific heating rate, it can be said that higher values are observed for the alloy with Pt. Therefore, we can expect a larger number of nuclei for the Mg_72_Zn_27_Pt_1_ alloy, and therefore a more fine-grained structure compared to Mg_72_Zn_27_Ag_1_.

This means that the morphology of crystal structures resulting from non-isothermal crystallization, i.e., the number of individual forms, depends on the processing conditions. The decisive factor is the heating speed. By making appropriate changes to the heating rate, the morphology of the composite based on the amorphous Mg_72_Zn_27_Pt_1_ or Mg_72_Zn_27_Ag_1_ alloy reinforced with a crystalline phase can be determined in a specific way.

The constant *k* gives information about the rate of the crystallization process. Both alloys have values of similar order for each heating rate, but, in most cases, the values for the alloy with Ag are slightly higher, which confirms its lower thermal stability and tendency for a faster crystallization process.

**Figure 9:**
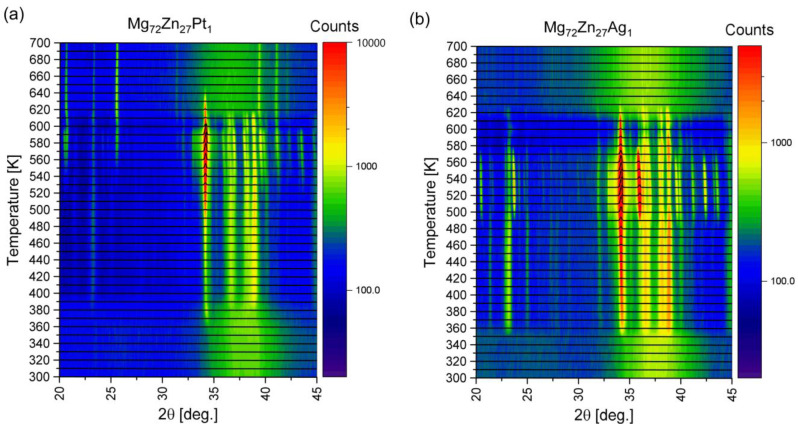
XRD patterns as a function of temperature for (**a**) the Mg_72_Zn_27_Pt_1_ alloy and (**b**) the Mg_72_Zn_27_Ag_1_ alloy.

X-ray diffraction was performed while heating amorphous ribbons from room temperature to 700 K (427 °C) at a heating rate of 5 K/min and data recording every 10°. Peaks corresponding to the crystalline phases that appear first in the process were identified. The reflections corresponding to the α-Mg phase (about 2*θ* = 34) and the Mg_12_Zn_13_ (in two places about 2*θ* = 37 and 2*θ* = 39) were recognized for both the Mg_72_Zn_27_Pt_1_ (Figure 9a) and Mg_72_Zn_27_Ag_1_ (Figure 9b) alloys. The crystalline phases formed first are the same as in the case of an Mg_72_Zn_28_ alloy [20].

The reflections from α-Mg have a much higher intensity compared to Mg_12_Zn_13_, which means that there is more of this first phase in the system. This is especially visible for the alloy with Pt, while the alloy with Ag shows slightly more Mg_12_Zn_13_ phase compared to Mg_72_Zn_27_Pt_1_. The lowest intensity for the Mg_12_Zn_13_ phase of these three alloys occurred for the two-component alloy [20].

Furthermore, the first peaks are observed around temperatures of 370 K and 350 K for Mg_72_Zn_27_Pt_1_ and Mg_72_Zn_27_Ag_1,_ respectively. Therefore, XRD also shows that the addition of Pt shifts the onset of crystallization towards higher temperatures compared to the binary alloys Mg_72_Zn_28_ [20] and Mg_72_Zn_27_Ag_1_.

The visible difference in the value of the transformation onset temperature obtained using DSC and XRD results from the measurement procedure. In the case of the measurement made using XRD, the measurement procedure while heating the amorphous ribbon from a temperature of 300 K to 700 K at a speed of 5 K/min was as follows: the device heats the ribbon at a speed of 5 K/min, and then stops every 5 K and measures for a period of 20 min. Following this measurement time of an amorphous ribbon at a specific temperature, the nucleation of the crystalline phase may occur. The situation is different in the case of DSC, where the measurement was made online. Therefore, the XRD results should be treated as results showing the nature of the transformation (e.g., the sigmoidal shape of the transformation) and the phase components of the structure that appear when the ribbon is heated.

## 4. Conclusions

The amorphous ribbons of the alloys Mg_72_Zn_27_Pt_1_ and Mg_72_Zn_27_Ag_1_ were successfully manufactured. The DSC and XRD investigations allow the following conclusions to be drawn:

- The addition of platinum and silver causes crystallization with one distinct crystallization peak and shifts the onset of crystallization towards higher temperatures compared to the two-component Mg_72_Zn_28_ alloy [20], but Pt has the greatest influence;
- The crystallization in the Mg_72_Zn_27_Ag_1_ alloy occurs faster than in the Mg_72_Zn_27_Pt_1_ alloy, and the Mg_72_Zn_27_Pt_1_ alloy has higher (temporary) thermal stability due to the difference in the size of the atoms of all alloy components;
- The crystallization in Mg_72_Zn_27_Pt_1_ and Mg_72_Zn_27_Ag_1_ is mainly based on the growth of a single and a two-dimensional crystal and the constant/decreasing nucleation rate during the process;
- The higher values of *n* for Mg_72_Zn_27_Pt_1_ indicate a greater number of nuclei and grains and, therefore, a more fine-grained structure;
- The XRD tests indicate the presence of α-Mg and Mg_12_Zn_13_ phases for both Mg_72_Zn_27_Pt_1_ and Mg_72_Zn_27_Ag_1_, as in the case of a two-component Mg_72_Zn_28_ alloy [20];
- The phase contribution is the greatest for Mg_72_Zn_27_Ag_1_, but for Mg_72_Zn_27_Pt_1_, it is also greater than for the binary Mg_72_Zn_28_ alloy [20].

## Figures and Tables

**Table 1:** Designated characteristic temperatures for amorphous Mg_72_Zn_27_Pt_1_ and Mg_72_Zn_27_Ag_1_ alloys heated at different rates in DSC.

Alloy	*β* [K/min]	τ [min]	$T_{g}$ [K]	$T_{x}$ [K]	$T_{p}$ [K]	$T_{x\_end}$ [K]
Mg_72_Zn_27_Pt_1_	5	21.43	393.90	404.82	414.35	426.88
	10	11.98	397.69	411.85	421.45	437.47
	20	6.53	404.75	420.07	430.14	448.85
	40	3.18	415.29	429.50	440.50	461.86
	80	1.92	423.80	442.57	456.93	491.17
Mg_72_Zn_27_Ag_1_	5	13.26	375.89	378.07	384.09	390.32
	10	9.30	377.64	384.48	390.81	400.36
	20	4.31	387.39	392.82	399.14	413.26
	40	2.51	394.98	402.62	409.75	433.96
	80	1.57	410.98	417.19	429.41	481.76

**Table 2:** Values of activation energies calculated using the Kissinger method for Mg_72_Zn_27_Pt_1_ and Mg_72_Zn_27_Ag_1_ alloys.

Alloy	$E_{g}$ [kJ/mol]	$E_{x}$ [kJ/mol]	$E_{p}$ [kJ/mol]
Mg_72_Zn_27_Pt_1_	114.56	102.52	95.01
Mg_72_Zn_27_Ag_1_	88.60	85.78	76.38

**Table 3:** Non-isothermal crystallization kinetic parameters calculated using the Avrami–Jeziorny method for the Mg_72_Zn_27_Pt_1_ and Mg_72_Zn_27_Ag_1_ alloys.

Alloy	*β* [K/min]	n	k	$k_{c}$
Mg_72_Zn_27_Pt_1_	5	2.5	0.474	0.095
	10	2.4	0.820	0.082
	20	2.4	1.523	0.076
	40	2.3	2.603	0.065
	80	2.1	3.554	0.044
Mg_72_Zn_27_Ag_1_	5	2.6	0.764	0.153
	10	2.5	1.281	0.128
	20	2.3	2.185	0.109
	40	2.0	3.182	0.080
	80	1.7	3.122	0.039

## Data Availability

The data presented in this study are available on request from the authors—A.P. pierwola@agh.edu.pl and J.L. lelito@agh.edu.pl.
